# Long COVID Researchers Are on the Hunt for Causes and Cures

**DOI:** 10.1021/acscentsci.3c01331

**Published:** 2023-11-02

**Authors:** Shi En Kim

Hannah Davis misses her old self. Like so many people around the world, she has seen her life upended by long COVID, which has made many once-routine activities impossible. The 32-year-old has stopped working at her job in the field of machine learning and generative models. It’s too cognitively taxing; the lights from display monitors are disorienting. Merely standing up from a sitting position causes her heart rate to shoot to 170 beats per minute, the equivalent of doing a good jog.

**Figure:**
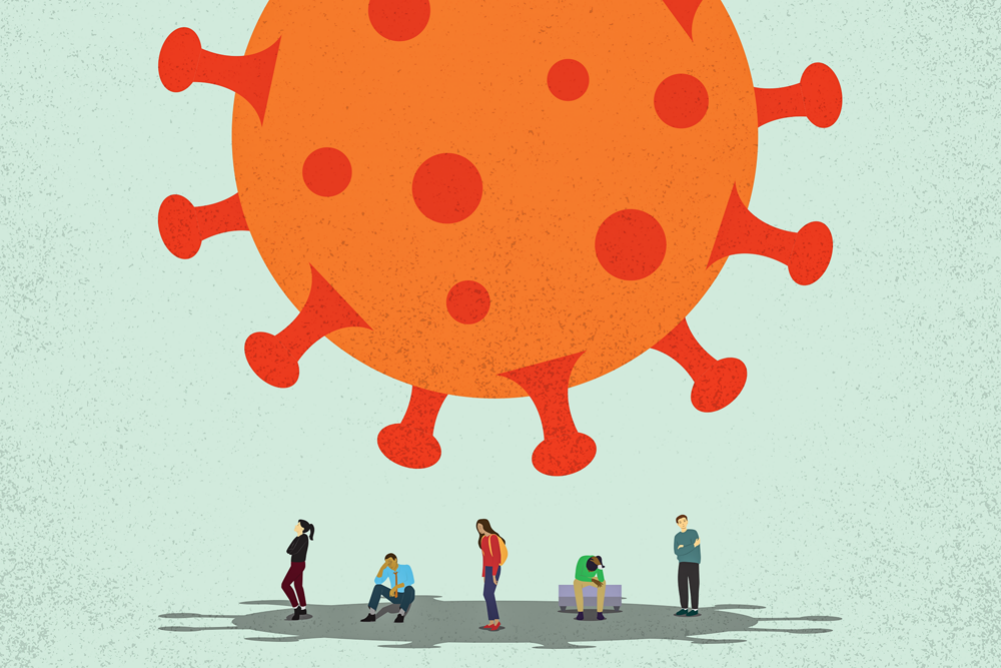
Credit: Will Ludwig/*C&EN*/Shutterstock

“A phone that doesn’t hold a charge” is how Davis describes how she feels. “Keep plugging it in and keep plugging it in, and then it’ll just last 20 minutes.”

There are multiple terms for what Davis experiences: postacute sequelae of SARS-CoV-2, post-COVID conditions, chronic COVID, and long COVID—the most popular name. She is one of an estimated 65 million people worldwide still suffering from COVID-19’s fallout weeks to months after their initial infection with the virus SARS-CoV-2.

About 10% of those who contract the virus end up with several of long COVID-19’s 200 documented symptoms. The most debilitating of these include the prolonged physical exhaustion known as myalgic encephalomyelitis/chronic fatigue syndrome (ME/CFS); cognitive impairment; and postural orthostatic tachycardia syndrome (POTS), which involves the dysregulation of the body’s background systems, such as heart rate. Some eventually recover fully, but many are still grappling with the aftereffects.

As disruptive as her long COVID is, Davis hasn’t looked back, and she is taking charge of her own fate. Just months after the start of the pandemic, she and others with long COVID founded the Patient-Led Research Collaborative, an advocacy and research organization that seeks to accelerate new findings on long COVID and spread awareness about the latest treatment options.

So far, no treatment has been approved specifically for long COVID. And the syndrome is still an enigma to many researchers because of the sweeping physiological changes that accompany it. “There are so many different syndromes underneath that lead me to believe that there might be multiple underlying causes,” says physician William Q. Pittman, assistant director of the Long COVID Program at UCLA Health, part of the University of California, Los Angeles.

But thanks to tireless efforts by scientists and patients such as Davis, researchers are starting to illuminate the biological underpinnings of the disease, and candidate treatments are already advancing through the clinic. Over 300 long-COVID studies from research institutions and drug companies alike populate the U.S. government clinical trial registry. Those trials should provide more clues to how long COVID wreaks havoc in the body and how it might one day be countered.

## Viruses all the way down

One hypothesis for a root cause of long COVID is a lingering viral presence in the body after an infection. Studies have reported viral flotsam, such as RNA and protein fragments, in the respiratory tract, blood, gut, poop, and extracellular vesicles of long-COVID patients. Autopsies of people who died weeks to months after contracting COVID-19 also found viral detritus in the brain, muscles, reproductive organs, eyes, and lungs. In a recent report, researchers uncovered viral traces in the tongues of people who had lost their sense of taste for months after infection. But it isn’t clear whether these are long-term leftovers or evidence of a persistent infection.

Some researchers theorize that SARS-CoV-2 may remain in the body and continue to replicate long after the acute infection has passed. There’s precedent for such behavior. For example, the Ebola virus takes cover in sanctuary tissue, only to reemerge later. In one patient, researchers found a high concentration of RNA strands, which suggests that the virus might be replicating in semen.

Although viral replication of SARS-CoV-2 has not been directly observed in long-COVID patients, some researchers are not ruling it out. “The whole idea is that, likely, it’s deeply hidden,” says Linda Geng, a primary care physician at Stanford Health Care. “In living human beings, it’s difficult [for researchers] to access a lot of deeper tissues” to find these viruses, she explains. The autopsy studies demonstrate the pervasiveness of viral particles and give researchers reason to suspect that viruses could lurk throughout the body.

The possible presence of viral squatters that continue to drive infection has motivated researchers to investigate whether antivirals can treat long COVID. In an anecdotal report of four patients taking Paxlovid, Pfizer’s combination drug of ritonavir and the antiviral nirmatrelvir, three reported improvement in chronic symptoms. Four clinical trials in the U.S. and Europe are underway to investigate whether Paxlovid can ameliorate long-COVID symptoms and potentially improve quality of life.

“There is some evidence that Paxlovid decreases development of long COVID,” says Jai Marathe, an infectious disease specialist at Boston Medical Center, in an email to *C&EN*. Marathe isn’t involved in any of the clinical studies involving the drug.

If long COVID’s symptoms are elicited not by the replicating SARS-CoV-2 virus but by its RNA rubble, then going after these fragments makes for a sensible therapeutic strategy. Resolve Therapeutics has a drug candidate with this mechanism of action. RSLV-132 consists of an antibody-straddling ribonuclease that patrols the bloodstream to chew up any loose nucleic acids outside the cell, viral or not. It completed Phase 2 clinical trials for long COVID in March.

But a bulky protein drug has an obvious limitation: it’s too big to enter a cell. “If you want to degrade a particular RNA inside the cell, that approach would not work,” says Gonçalo Bernardes, a chemical biologist at the University of Cambridge.

His team’s solution is to give RNA busters a small-molecule makeover. The researchers discovered that a lightweight imidazole group is sufficient to initiate a degradation sequence on any RNA it covalently tags. To make sure the compound leaves other nucleic acids alone, the researchers attach their imidazole warhead to binder ligands with a penchant for structural motifs on the RNA of SARS-CoV-2.

Bernardes’s team has demonstrated that its molecule can decrease the viral load in COVID-infected mouse models. Now, the researchers are continuing their drug optimization and development efforts through a company in stealth mode. Their goal is a drug that can one day treat both acute infection and long COVID.

Long COVID could also involve more than one virus. Some researchers suggest that the syndrome stems from the reactivation of viruses that had infected the body previously. Common bugs such as human herpes viruses stay dormant in the body until another infection reawakens them. A COVID-19 infection could weaken the immune system to the extent that the body can no longer keep the preexisting viruses in check.

“Acute COVID illness is a little bit like a tsunami,” says Michael VanElzakker, a neuroscientist at Harvard Medical School. Just as a tsunami can level human-made structures and drastically reshape the landscape it inundates, so SARS-CoV-2 infection can change the milieu of the host, he says.

Before the pandemic, the Epstein–Barr virus (EBV) was linked to ME/CFS in a subset of patients. Researchers studying ME/CFS have found EBV-related antibodies in newly diagnosed individuals. In anonymized and placebo-controlled studies, the antiviral valganciclovir, which targets the herpes virus and EBV, improved fatigue levels and cognitive function in patients with ME/CFS. Since ME/CFS is a hallmark of long COVID and previous infection with EBV is one of the risk factors for it, EBV could be the common culprit.

Last year, the biotechnology company Virios Therapeutics initiated clinical studies on a different antiviral, valacyclovir, for long COVID. The open-label study demonstrated that the drug improved symptoms of fatigue, pain, and autonomic dysfunction among its 22 enrollees, all of whom were female.

## A forever war inside the body

Long COVID’s indications also include elevated levels of immune signaling proteins and a lack of naïve T and B immune cells. People diagnosed with long COVID often have an immune system on constant high alert.

A study showed that 60% of 55 long-COVID patients carried inflammatory biomarkers in their bloodstream. In one mouse study, the authors found that spike proteins from the virus facilitated infiltration past the blood-brain barrier. The chronic neuroinflammation in the wake of the virus’s pillaging may explain long COVID’s hallmarks of memory and attention loss.

It could be that the body’s defenses are simply not turning off, as if the immune system is still shadowboxing a viral ghost. Alternatively, the virus may still be present and driving inflammation, in line with the viral persistence theory. Either way, the constant immune activity contributes to debilitating malaise and can significantly cramp a person’s quality of life.

“When people talk about this large immune response, I usually use the word *inefficient*, rather than *exaggerated*, because...it’s not working,” VanElzakker says.

Immune dysregulation points toward another suspect behind long COVID: autoantibodies. These defense proteins are often found in the blood of people with severe or long COVID. Autoantibodies turn against the host they’re supposed to protect; while researchers don’t think they are the lone drivers of long COVID, they may be coconspirators, working in unison with inflammatory cofactors to bring about biological bedlam.

If so, anti-inflammatory medication may address long-COVID symptoms by quieting the overactive immune response. “It’s possible that in some cases, or even many cases, what you want is to get the immune system to relax and say, ‘Let’s just call a truce.’ ” VanElzakker says.

Enter naltrexone. While the drug is best known as an opioid antagonist, when given at low doses it reduces the levels of cytokines originating in the central nervous system and decreases neuroinflammation. Before the pandemic, physicians prescribed naltrexone to treat chronic pain and ME/CFS, conditions that overlap with long COVID. “This has been a known treatment for [ME/CFS] for a long time,” UCLA’s Pittman says. Although the exact biological mechanism still isn’t known, he calls efforts to repurpose the drug for long COVID “very reasonable.”

In one study, 36 long-COVID patients taking naltrexone reported improvements in their symptoms, including higher energy levels and better sleep. The drug is also undergoing a 160-person Phase 2 clinical study to gauge its efficacy for alleviating post-COVID fatigue.

**Figure:**
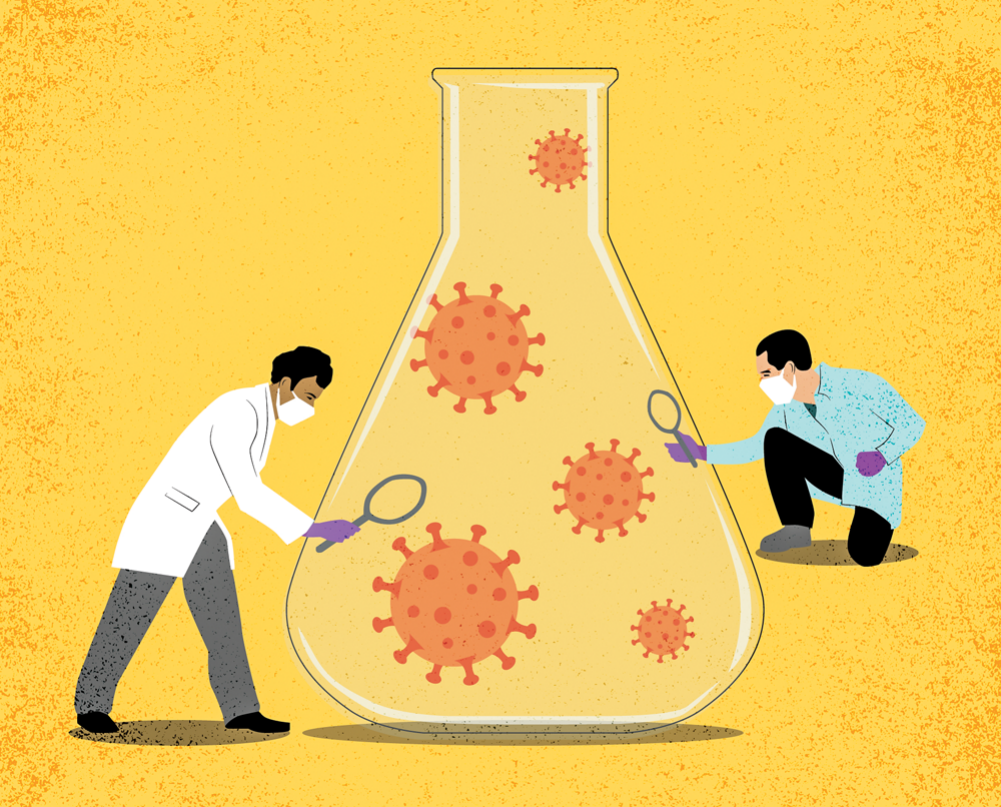
Credit: Will Ludwig/*C&EN*/Shutterstock

Another promising candidate for long COVID is aripiprazole, an antipsychotic drug approved for schizophrenia. The medication interacts strongly with dopamine and serotonin receptors in the brain to mediate neural signaling pathways. Like naltrexone, it can promote anti-inflammatory activity in the brain by elevating the levels of temperate cytokines over the combative kinds.

Aripiprazole has also already demonstrated some promise for treating ME/CFS: a retrospective study out of Stanford University showed that 77 of 101 patients self-reported improvement in fatigue, brain fog, and postexertional malaise after taking aripiprazole daily for several months. While no long-COVID-specific clinical trials of the drug are underway, physicians have prescribed it off label—that is, outside its officially approved use.

## It’s in the veins

Persistent inflammation in the blood vessels may also lead to another insidious effect: microclots made from clumps of fibrinogen and amyloid and reinforced with antiplasmin. Some researchers think these clots drive long COVID by clogging capillaries and starving tissues of oxygen—thus leading to fatigue, increased risk of stroke, and body aches. Antiplasmin gives microclots their abnormal longevity by preventing their natural solubilization. Adding fuel to the fire, hyperactive platelets prowl the bloodstream, boosting opportunities for microclot mischief by glomming onto the walls of blood vessels and restricting flow.

Researchers say microclots aren’t necessarily a primary cause of long COVID but rather a downstream consequence of viral remnants. “We’re not saying that thrombotic endotheliitis is the beginning and the end, but we are saying it is a prominent and specific part of the reason why we see long-COVID symptoms,” says Resia Pretorius, a professor in the physiological sciences at Stellenbosch University.

Pretorius was part of a team that inspected microclots for their composition. Inflammation from SARS-CoV-2’s spike proteins commonly results in endothelial damage, which leads to pathologic clotting. Clotting in turn can perpetuate further inflammation and blood vessel damage. Drugs that disrupt at any stage of this vicious cycle, such as by preventing clot formation, could let tissues heal once and for all.

Because COVID-19 increases the risk of thrombosis among patients in the hospital, they often receive anticoagulants such as heparin during their stay. In an open-label, placebo-controlled trial with 318 participants, researchers in Brazil investigated whether the practice should be continued after patients are discharged. They found that a daily dose of the anticoagulant rivaroxaban for 35 days posthospitalization led to a lower rate of thrombosis.

The study didn’t name long COVID per se, but research is already underway to cement the connections between blood clots and the condition and its treatability. STIMULATE-ICP, a 4,500-patient study focused on long COVID and sponsored by University College London, aims to examine the efficacy of drugs that have gained a foothold in the patient community but without clinical data to show for it. Those drugs include rivaroxaban. The researchers will announce their first results by the end of this month.

Some researchers are pushing a more drastic “triple therapy” to weed out clots. In 2021, Pretorius’s team tested a monthlong regimen of double antiplatelets and the anticoagulant apixaban among 24 people with long COVID. According to a preprint, all the participants found complete relief from their fatigue.

As promising as they sound, blood thinners have inherent risks. “Anticoagulants can have a side effect of bleeding, which can be very dangerous,” says Petter Brodin, a pediatrician and immunologist at the Karolinska Institute and Imperial College London. “We need to be very careful and make sure that we know what we’re doing.”

## Restoring energy levels

A growing body of research also suggests that COVID-19 may also be a disease of mitochondria, of metabolic homeostasis gone awry. “Mitochondrial dysfunction can explain a lot of the symptoms” of long COVID, says Akiko Iwasaki, an immunobiologist at Yale University. “It’s literally like not fueling your battery.”

The damage that SARS-CoV-2 inflicts on the vital organelles is manifold: it can hijack mitochondria to inhibit gene expression, reduce membrane potential, toggle them to a sluggish metabolic pathway, inhibit energy transduction, and eventually cause organelle death. In the aftermath of an acute infection, the detritus of free-floating mitochondrial DNA circulates in the blood like scattered bank notes at a crime scene.

Mitochondria aren’t just the energy reactors of the cell but gatekeepers of the immune system. They are hubs of metabolic processes that inadvertently generate reactive oxygen species, but they also produce key enzymes to neutralize those harmful oxidizers and limit their damage before they pinball around the cell. As SARS-CoV-2 tampers with these abilities, the body careens toward constant inflammation and a diminished ability to generate energy.

Mitochondria’s role in long COVID is still somewhat speculative, and the treatments that target these organelles are even more so. But several mitochondria-mitigating molecules have been observed to reduce the severity of COVID-19 infection. They are also a good starting point for developing potential treatments for long COVID.

Coenzyme Q10 (CoQ10), or ubiquinone, an antioxidant that occurs naturally in the body, has been explored as a supplement for people with ME/CFS. The compound has also found its way into long-COVID studies, but a 121-person study in Denmark found that a 6-week regimen of CoQ10 did not significantly improve fatigue levels compared with the placebo.

Recognizing the overlapping biology at play, the biotech firm Axcella Therapeutics recently pivoted from treating nonalcoholic steatohepatitis liver disease to addressing chronic fatigue in long COVID. Axcella’s investigational supplement, AXA1125, is a concoction of six amino acids in concentrations higher than those found in the average protein shake. While their exact mechanism is still unknown, these endogenous metabolic modulators purportedly regulate various biological processes after the body metabolizes them into diverse compounds that modify multiple pathways.

“We don’t have a precise target,” says Margaret Koziel, Axcella’s former chief medical officer. “We’re going after multiple pathways at the same time.”

A multipronged therapeutic approach may be fitting for such a multifaceted disease. A 41-person randomized Phase 2a trial showed that while the supplement failed to improve the biological markers for mitochondrial health, participants reported feeling less fatigued. Axcella is now getting ready for a follow-up Phase 2b study.

Like microclots, mitochondrial mayhem may be a downstream driver of long COVID but not its root cause. “Whether or not these supplements can restore the function of mitochondria is unclear,” Yale’s Iwasaki says. “You might have a transient improvement, but this is not going to be a solution long term.”

## Prevention over cures

Some research has demonstrated that reducing the severity of COVID-19 at the height of infection aids in preventing long COVID down the line. Treating people with antivirals or other drugs when the symptoms appear may help with both present and future conditions. Ziyad Al-Aly, a clinical epidemiologist at the Veterans Affairs St. Louis Health Care System, calls it “hitting the virus hard and early with antivirals,” just like “suppressing a fire.”

A retrospective study identified that people who took Paxlovid within 5 days of a positive test were 26% less likely—or had a 4.5% reduction in absolute risk—to become COVID-19 long-haulers than those who went without. The antiviral also whittled the odds of hospitalization and death when the patients were in the throes of a viral assault.

One nonantiviral drug seems to work similarly, by reducing illness and warding off long COVID. Metformin, a diabetes drug, made its way into the world of COVID-19 when researchers at the University of Minnesota Medical Center studied whether it could improve infection outcomes among hospitalized people.

The researchers later rolled their study into a Phase 3 trial for long-COVID prevention by following up with participants for 300 days. They found that metformin reduced long-COVID incidence by 41% and absolute risk by 4.1% among people who took the drug after the onset of symptoms. Previous research had also found that metformin inhibited SARS-CoV-2 replication in cell cultures and reduced viral load in patients. Whatever the mechanism, experts agree that the best thing about metformin is that it’s cheap and proven safe.

Arguably, the best tool for preventing long COVID is our all-around best protection against COVID-19: vaccines. Multiple studies have shown that a vaccinated person who later catches COVID-19 has a lower chance of developing chronic symptoms. Moreover, the vaccine’s preventative power increases with booster shots. But the data are split on whether or not receiving a vaccine after infection can ease long-COVID symptoms.

Just as vaccinated people are susceptible to breakthrough infections, so vaccines don’t fully shield against long COVID. Even if a person gets away scot-free after the first infection, they still face the risk of long COVID if they’re reinfected. People with mild COVID-19 can also develop chronic symptoms later. “One of the biggest misperceptions is that vaccination or prior infection protects you, or that you’ll react the same to every COVID infection,” Davis warns. “That’s not true.”

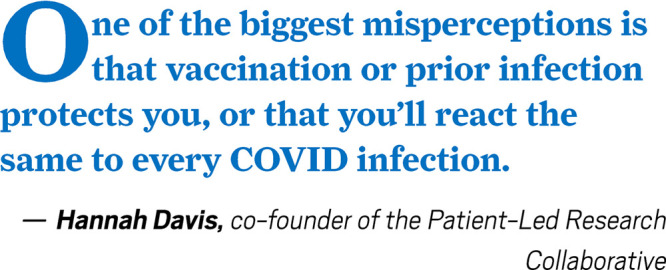



No preventative therapy eliminates the risk of developing chronic symptoms after a viral attack. The surest safeguard is to not get infected in the first place. “There’s no long COVID without COVID,” Al-Aly says.

## A long shot

Work continues to find long COVID’s causes and cures. But for now, patients get by with over-the-counter drugs that provide symptomatic relief. For example, antihistamines, a common allergy medication, may tackle inflammation and alleviate some discomfort. Long-hauler Davis also takes beta-blockers to deal with POTS. She says they are effective at alleviating some of her symptoms, but she knows that she’ll pay for her relief—her energy levels will crash in a few hours, making her feel worse than before.

“They’re a Band-Aid,” Davis says, “not cures.” And they often come with barriers in cost and coverage. In her own experience, she adds, “insurers will generally put up a fight.”

People can’t always count on clinical trials to get the latest cutting-edge treatment. For example, geographical access to trials can be a challenge. Even those who live near a clinical site have to muster their limited energy to show up. Davis says some people don’t bother registering for clinical trials because of their placebo-controlled and blinded format. No one wants to be in the group receiving a dud, she says. Instead, she advocates for crossover trials, in which all participants will rotate into the treatment cohort and eventually receive the therapy under investigation.

Without approved treatments or official diagnostics available, doctors have to rely on their experience and discussions with colleagues to provide care to long-COVID patients. For example, UCLA’s Pittman says he has prescribed the blood pressure medicine guanfacine for treating brain fog. His judgment stems from anecdotal evidence from Yale Medicine that the drug brought improvement among the hospital’s own patients with similar conditions.

Al-Aly at Veterans Affairs argues that treatment-seeking efforts could be more organized and systematic. “I think it’s kind of like the Wild West,” he says. “There is really no coherent approach.”

For some researchers, the scattershot strategy has advantages, especially for stirring up creativity and out-of-the-box thinking. But open-mindedness needs to be grounded in a scientific rationale and be backed by scientific rigor. “I don’t recommend anyone, doctor or patient, to just try things left and right,” Brodin at Karolinska Institute Brodin warns. “There’s a huge risk of possible side effects that might actually do more harm than good.”

The lack of a definition for long COVID also makes it challenging to find treatments. “That inability to have a uniform definition means we can’t know who the proper patients are to get treated,” says Panagis Galiatsatos, a pulmonary physician at Johns Hopkins Medicine. The vague terminology only highlights the lack of clarity around the disease pathology, which results in what he calls a “shooting from the hips” approach in both treatment and clinical studies.

The workaround is to define the target population very narrowly, stipulating the symptoms, time of infection, persistence of the condition, infection severity, and vaccination status in the eligibility criteria for an experimental treatment. If a treatment succeeds in clinical trials, doctors can say then who it is best suited to.

Some large-scale efforts are underway to understand the full-picture pathology of long COVID. They include the COVID Human Genetic Effort, a research consortium that aims to parse the genomic and immunological secrets behind why only some people get critically infected or develop long COVID.

The RECOVER Initiative by the U.S. National Institutes of Health aims to demystify long COVID in the name of basic research and clinical care. The program has received $1 billion in funding and boasts a large, diverse patient enrollment, but it has drawn criticism for its glacial progress.

Meanwhile, long COVID patients are taking action themselves—documenting their symptoms and collating research on their conditions. Amplifying these individual efforts are patient-led initiatives like the one Davis leads, so that the wealth of lived experiences can reach medical researchers and funders.

“What makes this postviral illness different from other ones is the scale and speed of communication” among the patient community, Davis says. Many people are living with the illness, and they would rather not sit around and wait.

## Shi En Kim is a freelance science writer

. *A**version of this story**first appeared in Chemical & Engineering News, an independent news outlet of the American Chemical Society*.

